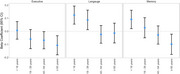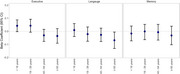# Age at First Traumatic Brain Injury and Domain‐Specific Cognitive Performance

**DOI:** 10.1002/alz70860_098288

**Published:** 2025-12-23

**Authors:** Holly C Elser, James Russell Pike, Rogan Magee, Rebecca F. Gottesman, Thomas H. Mosley, Eddie B Lee, David A. Wolk, Andrea L.C. Schneider

**Affiliations:** ^1^ Hospital of the University of Pennsylvania, Philadelphia, PA, USA; ^2^ Departments of Population Health and Medicine, New York University Grossman School of Medicine, New York, NY, USA; ^3^ Department of Neurology, Perelman School of Medicine, University of Pennsylvania, Philadelphia, PA, USA; ^4^ National Institute of Neurological Disorders & Stroke, Bethesda, MD, USA; ^5^ University of Mississippi Medical Center, The MIND Center, Jackson, MS, USA; ^6^ Perelman School of Medicine at the University of Pennsylvania, Philadelphia, PA, USA; ^7^ Center for Neurodegenerative Disease Research, Perelman School of Medicine, University of Pennsylvania, Philadelphia, PA, USA; ^8^ University of Pennsylvania Perelman School of Medicine, Philadelphia, PA, USA

## Abstract

**Background:**

Prior research describes short‐ and long‐term cognitive sequelae associated with traumatic brain injury (TBI). How age at first TBI influences cognition remains largely unknown.

**Method:**

In this prospective cohort‐study, time‐varying TBI (and age at first TBI: categorized as ≤18, 18‐39, 40‐64, ≥65 years) was defined by self‐report and diagnostic code from the *International Classification of Diseases*. Cognitive testing was performed at three visits for the Atherosclerosis Risk in Communities (ARIC) study between 2011–2019, including 11 tests which were combined into factor scores for memory, language, and executive function. Adjusted linear mixed‐effects models estimated the association of TBI with baseline domain‐specific cognitive performance and five‐year change overall and within strata defined by age‐at‐TBI. Models adjusted for baseline age, sex, race‐center, education, military veteran status, time‐varying alcohol use, and included an interaction between each covariate and time‐from baseline. Model coefficients are scaled to represent the standard deviation increase in factor scores for each respective cognitive domain. To mitigate bias potentially introduced by informative attrition, we used multiple imputation with chained equations with auxiliary variables to impute pre‐death cognitive scores among participants with missing outcome data.

**Result:**

We included 5,697 participants in our analysis, of whom 1,916 had a lifetime history of TBI (33.6%). At baseline, we observed the most pronounced difference between persons with and without TBI in executive function. Across domains we observed diminished cognitive performance among those age ≥65 years at the time of first TBI versus those with no TBI (Memory:‐0.099, 95%CI:‐0.177,‐0.022; Language:‐0.014, 95%CI:‐0.089,0.062; Executive Function:‐0.107, 95%CI: ‐0.180,‐0.034) (Figure 1). Differences in the 5‐year change in domain‐specific cognitive performance were more modest, but were also most pronounced for those with TBI at age ≥65 years, particularly for language (Memory:‐0.032, 95%CI:‐0.103,0.039; Language:‐0.069, 95%CI:‐0.131,‐0.006; Executive Function:‐0.036, 95%CI:‐0.091,0.020) although estimates were imprecise as evidenced by wider confidence intervals (Figure 2).

**Conclusion:**

In this study of community‐dwelling older adults, we observed domain‐specific cognitive impairment at baseline and over time among individuals with first TBI occurring at an older age.